# Voter-like Dynamics with Conflicting Preferences on Modular Networks

**DOI:** 10.3390/e25060838

**Published:** 2023-05-24

**Authors:** Filippo Zimmaro, Pierluigi Contucci, János Kertész

**Affiliations:** 1 Department of Computer Science, University of Pisa, 56126 Pisa, Italy; 2 Department of Mathematics, University of Bologna, 40126 Bologna, Italy; 3 Department of Network and Data Science, Central European University, 1100 Vienna, Austria

**Keywords:** sociophysics, opinion dynamics, agent-based models, networks

## Abstract

Two of the main factors shaping an individual’s opinion are social coordination and personal preferences, or personal biases. To understand the role of those and that of the topology of the network of interactions, we study an extension of the voter model proposed by Masuda and Redner (2011), where the agents are divided into two populations with opposite preferences. We consider a modular graph with two communities that reflect the bias assignment, modeling the phenomenon of epistemic bubbles. We analyze the models by approximate analytical methods and by simulations. Depending on the network and the biases’ strengths, the system can either reach a consensus or a polarized state, in which the two populations stabilize to different average opinions. The modular structure generally has the effect of increasing both the degree of polarization and its range in the space of parameters. When the difference in the bias strengths between the populations is large, the success of the very committed group in imposing its preferred opinion onto the other one depends largely on the level of segregation of the latter population, while the dependency on the topological structure of the former is negligible. We compare the simple mean-field approach with the pair approximation and test the goodness of the mean-field predictions on a real network.

## 1. Introduction

The formation of people’s opinions, choices and decisions is subject to social pressure: it is a general observation that in society, individuals (agents) take into account the behavior of others [1]. This aspect is at the root of most agent-based models of opinion dynamics [2,3,4,5]. Many such models have been introduced with the aim to understand the effects of different microscopic mechanisms of the opinion formation process [6,7]. Here, we consider the voter model [8,9,10], characterized by a simple imitative mechanism, introducing personal preferences attached to single individuals [11,12].

Prejudices or personal preferences generally come from the history of the individual (e.g., ideologies and partisanship [13]) and are assumed to evolve on a much longer time-scale than that of the opinion influencing interactions; in other words, they can be considered as fixed (quenched) features of the nodes throughout the dynamicsEven though this bias is a characteristic of the node, it is fundamentally different from other kinds of biases, such as the confirmation or algorithmic ones [14,15,16], because those are dependent on the node’s current opinion.

In a model with social pressure and quenched preferences, each individual is subjected to two “forces”: one inducing the individual to minimize conflicts with his neighbors, and the other exhorting the individual to stick to their own prejudice. A strong social pressure may lead the agent to adopt a public opinion in dissonance with their prejudices, a phenomenon which is defined as *preference falsification* in [17]. Otherwise, if the personal bias is strong, the individual may reject social coordination, accept conflicts more easily and stick to his prior view even if he finds himself in disagreement with many of his neighbors. The latter mechanism, when conflicting preferences are present among the individuals, may contribute to the emergence of *polarization* [18,19].

In this work, we generally consider two groups of interacting individuals with opposite preferences of different intensities. We focus in particular on the role of the social network, considering a modular network with two communities corresponding to the bias assignment. The network model mimics the realistic setting of two *epistemic bubbles*, where the agents with the same ideology share more links among themselves than with the other oppositely biased community. The result is an unbalanced choice of sources by the agent, who is systematically more influenced by his own community than by the other. In this paper, our main focus resides in determining the level of polarization between the two groups once the system has reached the stationary state, as a function of the preferences’ intensities and of the topological structure of the social network [20,21].

### 1.1. Literature Review

There is an analogy between the binary opinion dynamics models and the Ising model of statistical physics, where the personal preference can be achieved by a site-dependent external magnetic field. Following this analogy, we will characterize the opinion state of the individuals by a σ∈{+1,−1} “spin” variable. We study the so-called *partisanship voter model* (PVM), in which the preference toward one of the states modifies the transition rates of the voter dynamics accordingly and breaks the original symmetry between the two opinions. This dynamic should be distinguished from that of another biased voter model that we call the *voter model with media interactions* (VMMI) [22], where the personal bias expresses how much an individuum follows a node with a fixed opinion state connected to everybody (the “medium”). In Table 1, we summarize the transition rates of the Ising model and the biased voter models for homogeneous populations, where all individuals have the same personal biases. The same models in the bipopulated versions [23,24] are defined and explained in Table 2.

The PVM with homogeneous preferences can be historically individuated as a specific case of the Abrams–Strogatz model for language death [25] and as an agent-based model in [26,27]. The generalization to multiple biases was first proposed by Masuda et al. in 2010 [11]. The authors of [28] focused in particular on the finite-size effects for low bias intensity. In the successive work [12], the model was generalized for different compositions of the system and preferences’ intensities. In [29], the same model was considered where just a fraction of individuals were biased. Let us also point out that the introduction of *zealots* [30,31,32], i.e., agents that never change opinion and just try to convince others, can be traced back to the analyzed model if we properly tune the bias associated to such agents (i.e., setting $h_{z}=\left\{+1,-1\right\}$ depending on the type of commitment). The problem of social pressure and conflicting preferences was also studied in evolutionary game theory [33,34,35], with a focus on network effects [36] and supported with various social experiments [37,38,39].

### 1.2. Contributions and Article Structure

Despite the advancements in understanding the dynamics of systems evolving with voter-like processes, the influence of the social network topology in the PVM with conflicting preferences remains unexplored. This paper aims to address this gap by examining the interplay between fixed individual preferences and homophilic network structures (epistemic bubbles). Indeed, we expect homophily to play a crucial role in mitigating the social cohesion induced by imitative dynamics, when conflicting preferences are present, possibly leading the system to a polarized asymptotic state.

In this work, we consider the model of Masuda and Redner in its most general version [12], which we refer to as the (bipopulated) *voter model with preferences* (VMP): because personal preferences can arise from various factors and partisanship is just one of them, we propose this renaming to enlarge the applicability of the model as well as to establish a stronger connection to analogous models in the literature, e.g., to asymmetric game-theoretical models. We generally consider two classes of agents with opposite preferences of different intensities. The VMP is defined in Section 2 and solved on the fully connected network in Section 3.1. In Section 3.2, we study the model on a bi-modular network and calculate the phase diagram as a function of the model parameters using a mean-field approach. In Section 3.3, we apply the pair approximation [40,41] to the model on the modular network and compare its predictions to the mean-field results for sparse graphs. In the remainder of this article, we study the model on a real network with high modularity, the Political Blogosphere of 2004 US elections [42], and test the goodness of the mean-field predictions of the stationary state in the case of equally intense but opposite personal biases.

## 2. Model

The VMP, i.e., the generalization of the PVM of Masuda et al. [11,12], is defined as follows: the system of *N* agents is divided into two populations, or *classes*, of sizes $N_{1}$ and $N_{2}=N-N_{1}$, the agents $i=1,...,N_{1}$ belonging to the first and the remaining $i=N_{1}+1,...,N$ to the second one, with $\alpha =\frac{N_{1}}{N}\in \left(0,1\right)$ the fraction of individuals of the first population. A bias $h_{i}\in \left[-1,1\right]$ is assigned to each agent *i*, according to his class: in our bipopulated case, we assign the same $h_{1}$ to the individuals of the first population, similarly $h_{2}$ to all the individuals belonging to the second one. Each node’s opinion is represented as a binary spin $\sigma_{i}=\left\{+1,-1\right\}$ for i=1,...,N. The dynamics obey the following rules:

- One node (agent) *i* is selected uniformly randomly.
- A neighbor *j* of node *i* is selected uniformly randomly.
- If *i* and *j* have opposite opinions, *i* takes the opinion of *j* with probability $\frac{1}{2}\left(1+\sigma_{j}h_{i}\right)$. Otherwise, nothing happens.
- Repeat the process until consensus or apparent stabilization is reached.

The dynamics are a generalization of the classical voter model—retrieved for $h_{i}=0,\;\forall i$—where the individual copies his neighbor’s opinion with a probability equal to $\frac{1}{2}$ (in the original voter model this probability is 1, but the factor $\frac{1}{2}$ just slows down the dynamics). The biases modify the transition probabilities, favoring the transition toward the direction of the bias and disfavoring the opposite one. It is easy to show that if in the bipopulated case both of the biases point toward the same direction, then an infinite system will always reach consensus at the preferred state. Thus, in the following, we will consider $h_{1}\geq 0$ and $h_{2}\leq 0$ so that the individuals of the first population tend to prefer the +1 state, while the ones of the second class are ideologically biased toward the −1 opinion.

## 3. Results

### 3.1. Fully Connected Network

First, we study the model, for simplicity, on the complete network. This setting was already investigated in [12]; however, we complete the analysis by calculating the polarization measure at the stationary state for any choice of the parameters.

We define

(1) $$\rho_{1}=\frac{\sum_{i=1}^{N_{1}}\frac{1+\sigma_{i}}{2}}{N}\in \left[0,\alpha \right]\;\;\;\;\;\;\;\;\;\;\;\rho_{2}=\frac{\sum_{i=N_{1}+1}^{N}\frac{1+\sigma_{i}}{2}}{N}\in \left[0,1-\alpha \right]$$

as the ratios between the number of first-class spins (respectively, second) in the current up +1 state and the total number of spins in the system. The system of coupled ordinary differential equations which describes the evolution of such dynamical variables can be written generally in terms of the global rates $R_{\pm 1/2}$:

(2) $$\left\{\begin{array}{c} \dot{\rho_{1}}=R_{+1}\left(\rho_{1},\rho_{2}\right)-R_{-1}\left(\rho_{1},\rho_{2}\right)\\ \dot{\rho_{2}}=R_{+2}\left(\rho_{1},\rho_{2}\right)-R_{-2}\left(\rho_{1},\rho_{2}\right) \end{array}\right.$$

For example, the global rate $R_{+1}$ represents the probability per unit time that, when the system is currently in state $\rho_{1},\rho_{2}$, a spin of the first class undergoes the transition −1→+1, increasing the density of the up spin of the first class of 1/N, $\rho_{1}\to \rho_{1}+\frac{1}{N}$. Considering a time unit corresponding to *N* steps, i.e., $\delta t=N^{-1}$, the transition rates for a fully connected network are

(3) $$\left\{\begin{array}{c} R_{+1}\left(\rho_{1},\rho_{2}\right)=\left(\alpha -\rho_{1}\right)\frac{1+h_{1}}{2}\left(\rho_{1}+\rho_{2}\right)\\ R_{-1}\left(\rho_{1},\rho_{2}\right)=\rho_{1}\frac{1-h_{1}}{2}\left(1-\rho_{1}-\rho_{2}\right)\\ R_{+2}\left(\rho_{1},\rho_{2}\right)=\left(1-\alpha -\rho_{2}\right)\frac{1+h_{2}}{2}\left(\rho_{1}+\rho_{2}\right)\\ R_{-2}\left(\rho_{1},\rho_{2}\right)=\rho_{2}\frac{1-h_{2}}{2}\left(1-\rho_{1}-\rho_{2}\right) \end{array}\right.$$

For example, in the first rate $R_{+1}$, the first term $\alpha -\rho_{1}$ is the probability of choosing uniformly randomly a spin of the first class currently in the down state, while $\rho_{1}+\rho_{2}$ is the probability of choosing a neighbor in state +1 in the complete network, and eventually $\frac{1+h_{1}}{2}$ is the probability of the transition, according to the model dynamics. Thus, we have the following mean-field equations:

(4) $$\left\{\begin{array}{c} \dot{\rho_{1}}=\frac{1}{2}\left[\left(\alpha -\rho_{1}\right)\left(1+h_{1}\right)\left(\rho_{1}+\rho_{2}\right)-\rho_{1}\left(1-h_{1}\right)\left(1-\left(\rho_{1}+\rho_{2}\right)\right)\right]\\ \dot{\rho_{2}}=\frac{1}{2}\left[\left(1-\alpha -\rho_{2}\right)\left(1+h_{2}\right)\left(\rho_{1}+\rho_{2}\right)-\rho_{2}\left(1-h_{2}\right)\left(1-\left(\rho_{1}+\rho_{2}\right)\right)\right] \end{array}\right.$$

For the complete network in the N→∞ limit, the mean-field equations represent exactly the evolution of the system and they can be applied, as an approximation, to other networks. Not considering structural or dynamical correlations, we expect them to still be accurate on a sufficiently dense network without specific structural features [15,43], such as an Erdós–Rényi random graph with a probability of linkage of O(1).

Localizing the fixed points $\left(\rho_{1}^{*},\rho_{2}^{*}\right)$ of the system (4) and characterizing their stability by the analysis of the corresponding Jacobian matrices reported in Appendix A.1, one finds [12] the following:

- The positive (α,1−α) (all up spins) and negative (0,0) (all down spins) consensus points are always fixed points, for any combination of the parameters $\alpha ,h_{1},h_{2}$.
- When the positive (or negative) consensus is stable, it is the only stable fixed point.
- When both the consensus fixed points are not stable, another fixed point with $\rho_{1}^{*},\rho_{2}^{*}\in \left(0,1\right)$ appears. Such a fixed point, when it exists, is always stable.

Defining the total density of up spin $\rho =\rho_{1}+\rho_{2}$ and $\Delta =\frac{\rho_{1}}{\alpha }-\frac{\rho_{2}}{1-\alpha }$, the polarization can be expressed as P=|Δ|. As a first contribution of this paper, we calculate that at the *impasse* or *polarized* state the average density of up spins and the polarization, respectively, read

(5) $$\begin{array}{cc} \; & \rho^{*}=\frac{1}{2}\left(\frac{h_{2}-h_{1}}{h_{1}h_{2}}\alpha -\frac{1-h_{1}}{h_{1}}\right) \end{array}$$

(6) $$\begin{array}{cc} \; & \Delta^{*}=\frac{1}{h_{1}-h_{2}}\left(1+\frac{\alpha^{2}h_{1}^{2}+{\left(1-\alpha \right)}^{2}h_{2}^{2}-h_{1}^{2}h_{2}^{2}}{2\alpha \left(1-\alpha \right)h_{1}h_{2}}\right) \end{array}$$

Moreover, by analyzing the Jacobian, one can localize the critical value of the parameters at which the transitions from negative consensus to polarization and from polarization to positive consensus occur: taking $h_{1},h_{2}$ fixed and letting α vary, we have that the critical points of the transitions above are, respectively, at

$$\begin{array}{cc} \; & \alpha_{c}^{-}=\left(1-h_{1}\right)\frac{h_{2}}{h_{2}-h_{1}}\\ \; & \alpha_{c}^{+}=\left(1+h_{1}\right)\frac{h_{2}}{h_{2}-h_{1}} \end{array}$$

**Figure 1:**
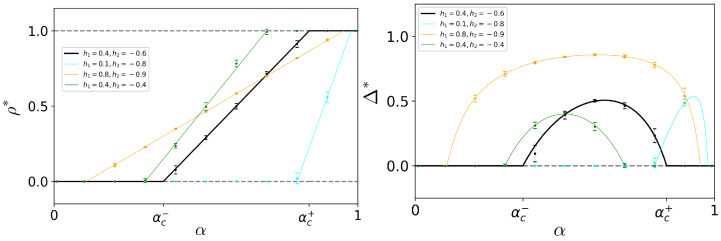
Bifurcation diagrams for the bipopulated voter model with preferences on the complete network. On the **left**, the total density of up spin is taken as order parameter; on the **right**, the polarization Δ is shown. The solid black line indicates the stable fixed point, while the dashed gray lines indicate the unstable ones, for the choice of the preferences’ intensities $h_{1}=0.4,h_{2}=-0.6$. The other solid colored lines locate just the coexistence stable fixed point for other choices of the intensities, as indicated in the legend. The points and their bars are, respectively, the average and the confidence interval of the order parameters calculated over 30 independent simulations of a system with 1000 agents, and they are reported in order to also test the validity of the mean-field treatment for relatively small system sizes.

The bifurcation diagrams, taking ρ and *P* as the order parameters and varying the composition α for various fixed $h_{1},h_{2}$, are shown in Figure 1: the bifurcation is of a transcritical type and the transitions are indeed continuous. The presented numerical simulations confirm that the analytical solutions work well for systems defined on relatively small, N=1000 complete graphs as well.

**Figure 2:**
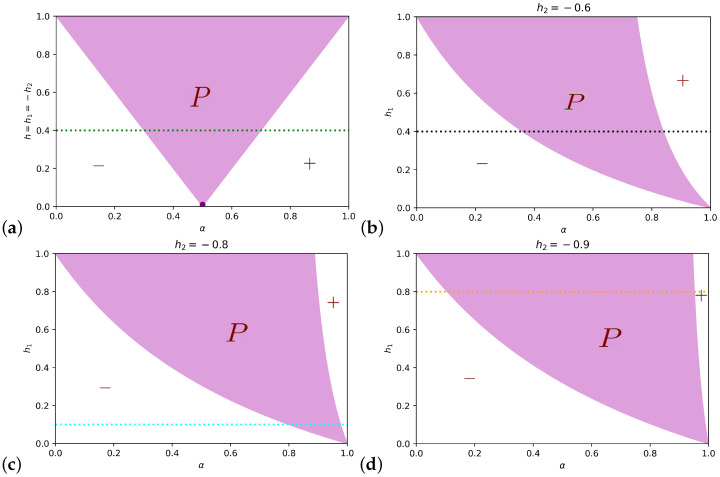
Phase diagrams for the complete network. The polarized area is colored in light purple and indicated with *P*, and in white are + and − consensus. (**a**) shows the mean-field phases in the αh plane, for equal and opposite preferences’ intensities $h_{1}=-h_{2}=h$. The dark purple dot represents the regime $\alpha =\frac{1}{2},h\to 0$ in which [28] have investigated finite-size effects. (**b**–**d**) report the phases in the $\alpha h_{1}$ plane, once fixed $h_{2}$, respectively, to −0.6,−0.8,−0.9. Each of the colored horizontal lines present in some of the plots represents the choice of the biases $h_{1},h_{2}$ as in Figure 1 (the colors correspond). They are reported in order to show how the lines intersect the different phases.

The length of the interval associated to the polarized state is $\alpha_{c}^{+}-\alpha_{c}^{-}=2\frac{h_{1}h_{2}}{h_{2}-h_{1}}$ which reduces to $\alpha_{c}^{+}-\alpha_{c}^{-}=h$ for $h_{1}=-h_{2}=h$. The phase diagram in this case (in the α,h plane) is shown in Figure 2a, while in the remaining plots of the figure $h_{2}$ is fixed to different values, and the phases in the plane $\alpha ,h_{1}$ are shown. To link the bifurcation and the phase diagrams, the horizontal lines corresponding to the choices of the biases in Figure 1 are reported in the latter.

Defining the critical mass of a population [44] as the minimum fraction of individuals of that population necessary to escape from consensus at the unpreferred opinion, we have that the critical masses of, respectively, the first and second populations are $\alpha_{c}^{-}$ and $1-\alpha_{c}^{+}$. For very low biases, the populations over the critical masses rapidly overturn the outcome of the system, switching the direction of consensus. In this model, the critical masses depend only on the biases and lay in the whole range (0,1).

### 3.2. Modular Networks

To study the effect of the topology reflecting the biased communities of the bipopulated VMP, we analyze the model on a network with two modules of sizes $N_{1}$ and $N_{2}$, generated by a stochastic block model (SBM) [45]. The SBM is defined by the intramodular ($p_{11},p_{22}$) and intermodular ($p_{12},p_{21}$) connectivities, i.e., the probabilities describing the corresponding linkings between the agents (for undirected networks $p_{12}=p_{21}$). We assume that the network results from homophilic interactions (epistemic bubbles) such that all agents within module 1 (2) have bias $h_{1}$ ($h_{2}$).

We start from the mean-field Equation (2) where the global transition rates are now functions of the connectivities of the block model:

(7) $$\left\{\begin{array}{c} R_{+1}\left(\rho_{1},\rho_{2}\right)=\frac{1}{\alpha p_{11}+\left(1-\alpha \right)p_{12}}\left(\alpha -\rho_{1}\right)\frac{1+h_{1}}{2}\left(p_{11}\rho_{1}+p_{12}\rho_{2}\right)\\ R_{-1}\left(\rho_{1},\rho_{2}\right)=\frac{1}{\alpha p_{11}+\left(1-\alpha \right)p_{12}}\rho_{1}\frac{1-h_{1}}{2}\left[p_{11}\left(\alpha -\rho_{1}\right)+p_{12}\left(1-\alpha -\rho_{2}\right)\right]\\ R_{+2}\left(\rho_{1},\rho_{2}\right)=\frac{1}{\alpha p_{12}+\left(1-\alpha \right)p_{22}}\left(1-\alpha -\rho_{2}\right)\frac{1+h_{2}}{2}\left(p_{12}\rho_{1}+p_{22}\rho_{2}\right)\\ R_{-2}\left(\rho_{1},\rho_{2}\right)=\frac{1}{\alpha p_{12}+\left(1-\alpha \right)p_{22}}\rho_{2}\frac{1-h_{2}}{2}\left[p_{12}\left(\alpha -\rho_{1}\right)+p_{22}\left(1-\alpha -\rho_{2}\right)\right] \end{array}\right.$$

For example, once a spin in the down state of class 1 is selected, the probability that we find one of its neighbors in the up state is $\frac{p_{11}\rho_{1}}{\alpha p_{11}+\left(1-\alpha \right)p_{12}}+\frac{p_{12}\rho_{2}}{\alpha p_{11}+\left(1-\alpha \right)p_{12}}$ (for more details, see Appendix B).

We end up with the mean-field evolution equations for the densities $\rho_{1},\rho_{2}$:

(8) $$\left\{\begin{array}{c} \dot{\rho_{1}}=C_{1}\left[\left(\alpha -\rho_{1}\right)\left(1+h_{1}\right)\left(p_{11}\rho_{1}+p_{12}\rho_{2}\right)-\rho_{1}\left(1-h_{1}\right)\left[p_{11}\left(\alpha -\rho_{1}\right)+p_{12}\left(1-\alpha -\rho_{2}\right)\right]\right]\\ \dot{\rho_{2}}=C_{2}\left[\left(1-\alpha -\rho_{2}\right)\left(1+h_{2}\right)\left(p_{12}\rho_{1}+p_{22}\rho_{2}\right)-\rho_{2}\left(1-h_{2}\right)\left[p_{12}\left(\alpha -\rho_{1}\right)+p_{22}\left(1-\alpha -\rho_{2}\right)\right]\right] \end{array}\right.$$

where $C_{1}=\frac{1}{2[\alpha p_{11}+\left(1-\alpha \right)p_{12}]}$, $C_{2}=\frac{1}{2[\alpha p_{12}+\left(1-\alpha \right)p_{22}]}$.

**Figure 3:**
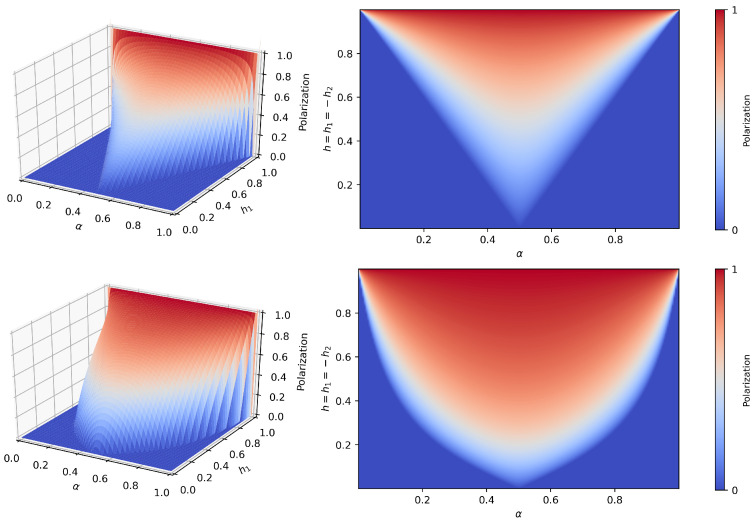
VMP on modular networks with equally strong opposite preferences, α,h plane. The mean-field predictions are shown, i.e., the stable fixed point of the system (8), for two choices of connectivities of the modular network. *Upper plots*: case $p_{11}=p_{22}=p_{12}=p_{21}=1$, corresponding to the fully connected topology (subplot (a) in Figure 2). *Lower plots*: case $p_{11}=p_{22}=1$, $p_{12}=p_{21}=0.3$, i.e., still symmetric probabilities but segregated communities. The polarization increases and the polarization area widens; nevertheless, the general qualitative behavior is preserved.

It is easy to see that the consensus states are still fixed points. The numerical study of the system shows that the qualitative behavior of the fully connected case is preserved, i.e., the different phases are separated by transcritical bifurcations, but the polarization area generally widens and the range of the polarized phase increases. Figure 3 compares the fully connected case and the topology characterized by two cliques ($p_{11}=p_{22}=1$) and intercommunity connectivities $p_{12}=p_{21}=0.3$, with equally strong opposite preferences $h_{1}=-h_{2}=h$. In general, the symmetric community structure decreases the values of the critical masses, with respect to the fully connected topology.

By considering the evolution of the normalized densities $\rho_{1}^{\prime }=\frac{\rho_{1}}{\alpha }\in \left[0,1\right]$, $\rho_{2}^{\prime }=\frac{\rho_{2}}{1-\alpha }\in \left[0,1\right]$ and defining the topological parameters

(9) $$\gamma_{1}=\frac{p_{12}\left(1-\alpha \right)}{p_{11}\alpha +p_{12}\left(1-\alpha \right)}\;\;\;\;\;\;\;\;\;\gamma_{2}=\frac{p_{21}\alpha }{p_{22}\left(1-\alpha \right)+p_{21}\alpha }$$

we have a consistent reduction in the number of parameters in the mean-field system on the SBM (8), which reads

(10) $$\left\{\begin{array}{c} \dot{\rho_{1}^{\prime }}=\frac{1}{2}\left[\left(1-\rho_{1}^{\prime }\right)\left(1+h_{1}\right)\left[\left(1-\gamma_{1}\right)\rho_{1}^{\prime }+\gamma_{1}\rho_{2}^{\prime }\right]-\rho_{1}^{\prime }\left(1-h_{1}\right)\left[\left(1-\gamma_{1}\right)\left(1-\rho_{1}^{\prime }\right)+\gamma_{1}\left(1-\rho_{2}^{\prime }\right)\right]\right]\\ \dot{\rho_{2}^{\prime }}=\frac{1}{2}\left[\left(1-\rho_{2}^{\prime }\right)\left(1+h_{2}\right)\left[\gamma_{2}\rho_{1}^{\prime }+\left(1-\gamma_{2}\right)\rho_{2}^{\prime }\right]-\rho_{2}^{\prime }\left(1-h_{2}\right)\left[\gamma_{2}\left(1-\rho_{1}^{\prime }\right)+\left(1-\gamma_{2}\right)\left(1-\rho_{2}^{\prime }\right)\right]\right] \end{array}\right.$$

The interpretation of $\gamma_{1},\gamma_{2}$ is straightforward in terms of the average internal and external degrees $z_{11},z_{12},z_{21},z_{22}$:

(11) $$\gamma_{1}=\frac{z_{12}}{z_{11}+z_{12}}\;\;\;\;\;\;\;\;\;\gamma_{2}=\frac{z_{21}}{z_{22}+z_{21}}$$

**Figure 4:**
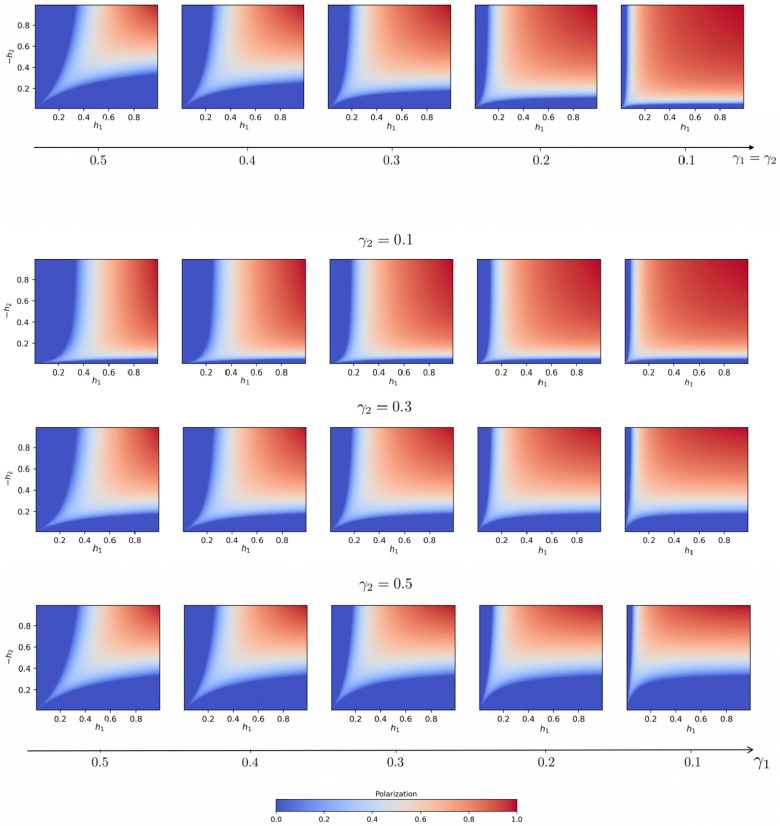
VMP on modular networks. Mean-field predictions for the polarization (Equation (10)) in the $h_{1},h_{2}$ plane for two equally open-minded communities ($\gamma_{1}=\gamma_{2}$, upper line), and for three values of $\gamma_{2}$, varying the open-mindedness of the first population $\gamma_{1}$. Notice that the open-mindedness decreases from left to right, meaning that moving to the right the groups become more and more segregated. Decreasing the open-mindedness, the consensus areas shrink and the system becomes more and more polarized.

Being the average fractions of external connections over the total number of connections of the agents in classes 1 and 2, $\gamma_{1},\gamma_{2}$ can be intended as the average *open-mindedness* of the individuals of, respectively, the first and second communities. Because in an epistemic bubble the agents overrepresent (i.e., are more linked to) their belonging community, we expect $\gamma_{1}\in \left(0,1-\alpha \right)$ and $\gamma_{2}\in \left(0,\alpha \right)$. The more far $\gamma_{1},\gamma_{2}$ are from these upper extremes, the more the individuals of the corresponding population have unbalanced sources of information, i.e., are trapped in the bubble. If the individuals have on average more connections within their belonging community rather than toward the other, then $\gamma_{1},\gamma_{2}\in \left(0,0.5\right)$. Figure 4 shows the stationary polarization $\Delta^{*}=\rho_{1}^{\prime *}-\rho_{2}^{\prime *}$ in the $h_{1}h_{2}$ plane, for different choices of the open-mindedness parameters $\gamma_{1},\gamma_{2}$, by numerically calculating the fixed points of the mean-field system (10) and determining their stability.

From the linear stability analysis of the consensus fixed points of the system (10), reported in Appendix A.2, we obtain the condition for the stability of the positive consensus

(12) $$\gamma_{1}h_{2}\left(1-h_{1}\right)+\gamma_{2}h_{1}\left(1-h_{2}\right)+2h_{1}h_{2}\geq 0$$

and for the negative one

(13) $$-\gamma_{1}h_{2}\left(1+h_{1}\right)-\gamma_{2}h_{1}\left(1+h_{2}\right)+2h_{1}h_{2}\geq 0$$

First, we fix $\gamma_{1},\gamma_{2}$ and determine the critical lines in the $h_{1},h_{2}$ plane. The line separating the space when the positive consensus is stable (below the line) and the one for which it is unstable reads

(14) $$-h_{2}^{c}\left(h_{1}\right)=\frac{\gamma_{2}h_{1}}{\gamma_{1}\left(1-h_{1}\right)+h_{1}\left(2-\gamma_{2}\right)}$$

and it is bounded superiorly by $-h_{2}^{c+}=\frac{\gamma_{2}}{2-\gamma_{2}}$, which is reached at $h_{1}=1$ and does not depend on $\gamma_{1}$.

**Figure 5:**
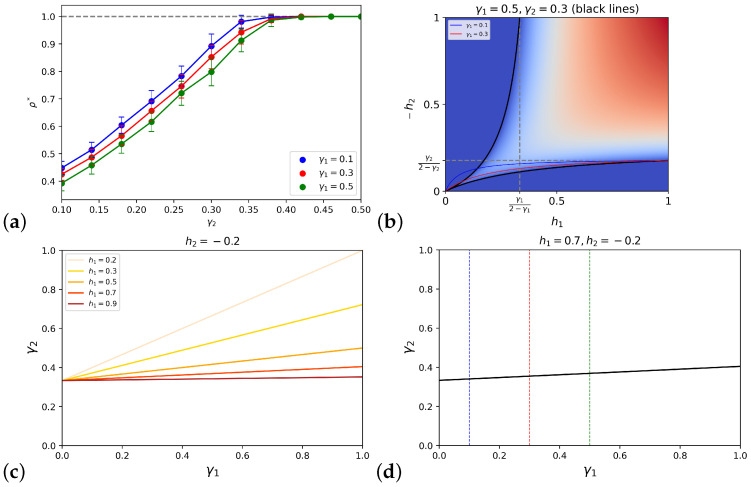
Simulations and analytical results for the VMP on a modular graph. (**a**) The average density of up spin at the stationary state is calculated over 30 runs of the model, with N=1000 agents and preferences $h_{1}=0.7,h_{2}=-0.2$, varying the open-mindedness of the second population and for 3 values of the open-mindedness of the first population, corresponding to different colors. We see that all three lines approach the positive consensus at approximately the same $\gamma_{2}$. (**b**) Critical lines for the positive and negative consensus points in the $h_{1},-h_{2}$ plane, for $\gamma_{1}=0.5,\gamma_{2}=0.3$ (black). In the background, the polarization values calculated numerically as in Figure 4 are reported, to show the consistency of the results of the linear stability analysis. The red and blue lines are the positive consensus critical lines for other values of $\gamma_{1}$, while $\gamma_{2}=0.3$ constantly. The figure is the analytical correspondent of $\gamma_{1}=0.1,0.3,0.5$, $\gamma_{2}=0.3$ plots in Figure 4. (**c**) Critical lines in the $\gamma_{1},\gamma_{2}$ plane for $h_{2}=-0.2$ and different $h_{1}$. The figure shows that the higher $h_{1}$, the more the critical line tends to a horizontal line. (**d**) Once the biases are fixed to $h_{1}=0.7,h_{2}=-0.2$, in the $\gamma_{1}\gamma_{2}$ plane the analytical critical line (16) and the values of $\gamma_{1}$ (vertical lines) corresponding to the lines in (**a**) are reported: we see that the intersections with the critical line (i.e., the points at which the positive consensus becomes stable) occur almost at the same $\gamma_{2}$, for all three values of $\gamma_{1}$.

In the same way, the critical line related to the negative consensus fixed point reads

(15) $$h_{1}^{c}\left(h_{2}\right)=\frac{-\gamma_{2}h_{2}}{\gamma_{2}\left(1+h_{2}\right)-h_{2}\left(2-\gamma_{1}\right)}$$

and it is bounded by $h_{1}^{c-}=\frac{\gamma_{1}}{2-\gamma_{1}}$, independent of $\gamma_{2}$. The critical lines are drawn for a choice of the open-mindedness parameters in Figure 5b.

In the $\gamma_{1},\gamma_{2}$ plane, the critical line for the positive consensus

(16) $$\gamma_{2}^{c}\left(\gamma_{1}\right)=\frac{-h_{2}\left(1-h_{1}\right)}{h_{1}\left(1-h_{2}\right)}\gamma_{1}-\frac{2h_{2}}{\left(1-h_{2}\right)}$$

is a straight line in the plane, whose coefficient tends to zero (the line flattens) for high $h_{1}$ and low $-h_{2}$. When this is the case, the critical points for different topologies of the first population, i.e., different $\gamma_{1}$, happen to be at approximately the same value of $\gamma_{2}$, as shown in Figure 5c,d. Naturally, symmetric considerations hold for the negative consensus.

The results of the linear stability analysis allow us to conclude that if one population is very committed or the other population’s bias is very low, e.g., when $h_{1}>>-h_{2}$, whether or not such a population manages to impose its preferred opinion depends largely on the open-mindedness of the other population $\gamma_{2}$, while the dependency on the topological structure of the committed population $\gamma_{1}$ is negligible.

We test this claim by numerically simulating the VMP on modular networks for a finite system of N=1000 agents, fixing $h_{1}=0.7,h_{2}=-0.2$ and varying the topology through the open-mindedness parameters $\gamma_{2}$ and $\gamma_{1}$. The results, shown in Figure 5a, support the mean-field predictions by showing that the critical value of $\gamma_{2}$ separating the polarization and positive consensus phases is approximately the same for all three lines in the $\gamma_{2},\rho^{*}$ plane, corresponding to the three chosen values of $\gamma_{1}$.

### 3.3. Pair Approximation

The aim of this section is to investigate how a more refined approximation better reproduces the model’s behavior on complex networks. We implement the so-called pair approximation (PA) [40,41], which takes into consideration dynamical correlations at a pairwise level.

For the sake of simplicity, the pair approximation is applied on an undirected regular and $m_{ij}$ neighbors of the population *j* currently in state +1.

The mean-field approximation consists of taking those probabilities highly peaked at the values of $\frac{m_{ij}}{z_{ij}}$ coinciding to the overall normalized densities $\rho_{1}^{\prime },\rho_{2}^{\prime }$, so having the shape of delta functions explicitly stated in ((A39)–(A42)). The pair approximation, instead, considers pairwise dynamical correlations by taking those probabilities as binomial distributions with single event probability corresponding to the ratio between the number of active links departing from the node’s type, intended as its class and state, and the number of connections from that node’s type. The argument is explained in the Appendix ((A43)–(A58)).

**Figure 6:**
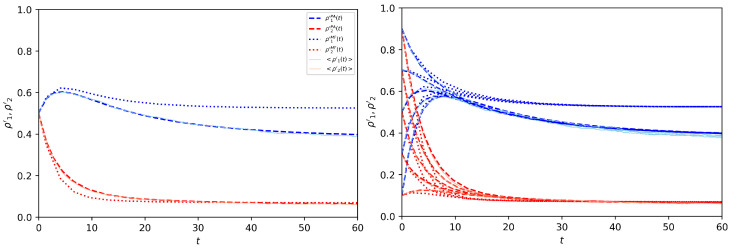
Pair and mean-field approximations. On the **left**, dynamics of the MF (dotted line) and PA (dashed lines) approximations compared with numerical simulations (thin solid lines), averaged over 30 independent runs. The graph is a sparse modular network of N=3000 nodes with two z− regular communities $z_{11}=4,z_{12}=2,z_{21}=1,z_{22}=3$; thus, $\alpha =\frac{2}{3}$ and $\gamma_{1}=0.4,\gamma_{2}=0.25$. The two communities’ preferences are, respectively, $h_{1}=0.3,h_{2}=-0.5$. The initial opinions are chosen uniformly random ($\rho_{1}^{\prime }\left(0\right)=\rho_{2}^{\prime }\left(0\right)=\frac{1}{2}$). On the **right**, same setting repeated for various initial conditions $\rho_{1}^{\prime }\left(0\right),\rho_{2}^{\prime }\left(0\right)$. The results show that even for low connectivities, the stable fixed point is unique.

The latter ratios are also dynamical variables of the system derived from the pair approximation (A59), which indeed consists of six coupled differential equations. The increase in complexity is justified by a gain in accuracy, consistent for low average degrees, i.e., for sparse graphs. In Figure 6, we compare the mean-field and pair approximation effectiveness in reproducing the dynamics of the system, on an extremely sparse modular network. We see that the gain in accuracy is consistent, and the PA is able to predict the dynamics almost perfectly. On the right plot of the figure, we test multiple initial conditions in order to check that, as in the mean-field treatment, the stable fixed point is unique.

### 3.4. Application to the Network of Blogs

**Figure 7:**
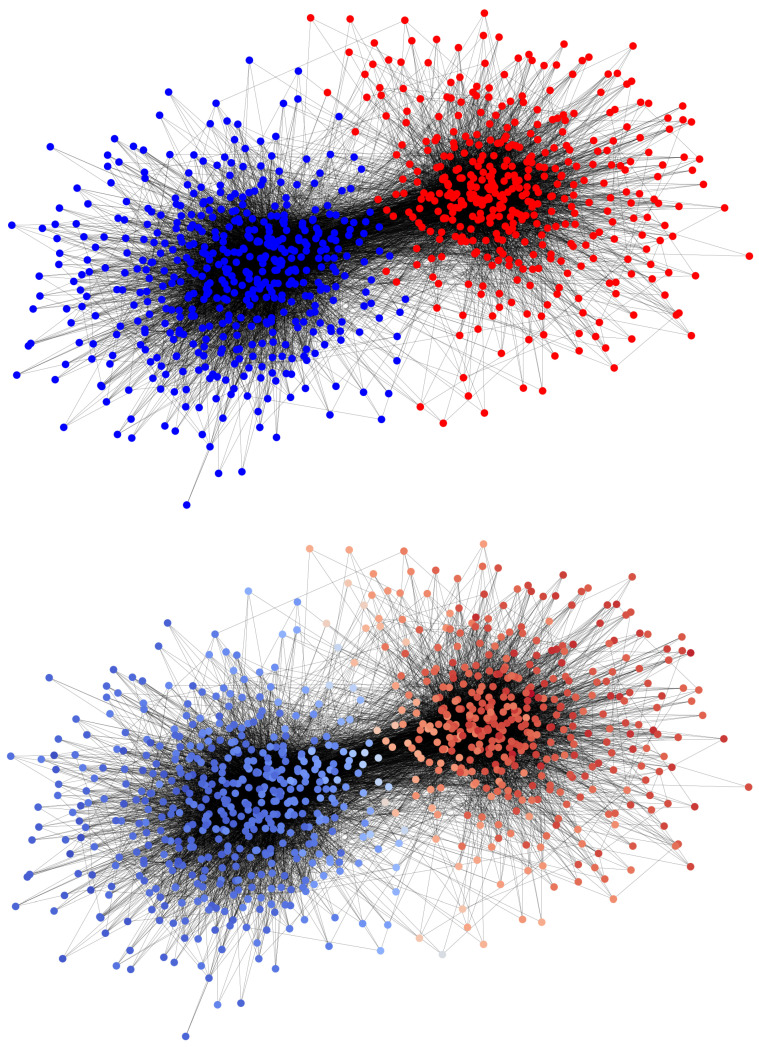
VMP on the 2004 Blogosphere. In the **upper** plot, the 2004 Political Blogosphere after community detection: nodes’ colors reflect political partisanship. In the **lower** plots, each node is colored according to the average state $\bar{\rho_{i}}$ at time t=50, for a bipopulated VMP where the populations are the ones computed by community detection and the preferences are set to $h_{r}=-h_{b}=0.2$. The colorscale goes from blue, corresponding to $\bar{\rho_{i}}=0$, to red ($\bar{\rho_{i}}=1$). The averages are computed running 100 independent simulations.

**Figure 8:**
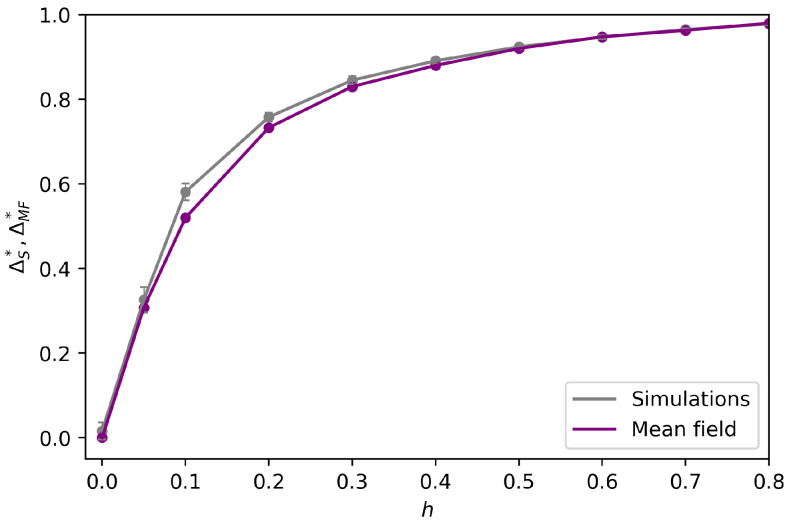
Mean-field predictions and simulations of the model on the 2004 Blogosphere. We compare the mean-field predictions (purple line) and the numerical simulations (gray line) of the asymptotic polarization on the 2004 Political Blogosphere, restricting to equally strong biases $h_{r}=-h_{b}=h$, for various *h*.

We run the bipopulated VMP on a real social network with a modular structure, to study how well the mean-field approximation performs on a real network with high modularity and potentially structural features as well as dynamical correlations. We take the network of Political Blogs during the 2004 American elections [42], characterized by the presence of two communities that reflect political bipartisanship. After eliminating the nodes with a degree less than 4, and transforming for simplicity the original directed network to undirected (such that $p_{12}=p_{21}$), we apply a community detection algorithm [46]. It turns out that the two communities, named “reds” and “blues” (shown in the upper plot of Figure 7), have sizes, respectively, $N_{r}=413$, $N_{b}=490$; average internal degrees $z_{rr}=34.02$, $z_{bb}=32.38$; and external degrees $z_{rb}=2.88$, $z_{br}=2.43$. We perform numerical simulations of the model, choosing, for simplicity, opposite and equally strong preferences ($h_{1}=-h_{2}=h$), for multiple values of *h*, and compare them to the mean field predictions on an SBM with the same average degrees. The results are reported in Figure 8: the mean-field predictions agree well with the empirical simulations, with a small gap emerging for weak preference intensities. In the lower plot of Figure 7, the average polarization of each node in the asymptotic state (t=50) over repeated runs of the model with equal and opposite biases fixed to h=0.3 is reported: as one could expect, the most open-minded nodes adopt their unpreferred opinion more frequently than the ones in the rest of their community. Despite this effect due to the heterogeneity of the degrees and specifically of the local open-mindedness, the mean-field predictions remain quite accurate.

## 4. Discussion and Conclusions

In this work, we have studied a system of individuals whose process of opinion formation is influenced by three factors: the imitative mechanism at the root of most of the models of opinion dynamics, the heterogeneous personal preferences of individuals for one opinion rather than the other and the homophilic phenomenon at the root of the so-called *epistemic bubbles*, which implies that each individual is more connected to individuals with the same preference and leads to the formation of a social network with a modular structure. We have considered two oppositely biased populations with preferences of different intensities, interacting through a modular network that reflects the phenomenon of epistemic bubbles, where two individuals with the same bias are more likely to get connected. Within the model’s framework, it turns out that in general an increasing segregation of the two communities of individuals with the same fixed preference induces and favors polarization. We have derived the system of differential equations governing the opinion dynamics within the mean-field and pair approximations. In the mean-field framework, we have analytically determined the conditions under which the individuals of one population manage to induce the whole system to converge to their preferred opinion. Moreover, we have shown that the achievement of consensus depends mostly on the topological structure of the “losing” population rather than on the one of the “winning” population. This disparity is more evident the greater the bias intensity of the “winning” group with respect to the “losing” one. In this sense, the model suggests that, in order to maintain consensus at their preferred opinion, it is convenient for the “winning” population to keep the level of segregation of the losing population under a certain threshold, also at the price of becoming slightly less segregated. In other words, it is convenient for the individuals of the “winning” population to establish a larger number of inter-class connections. On the other hand, the “losing” population must become more cohesive in order to escape from the consensus state at its unpreferred opinion.

The appropriateness of the assumptions at the basis of the abstract and minimalistic voter model with preferences needs to be tested in a real setting through the implementation of social experiments [5,47]. On the other hand, in perspective of an application of the VMP on real data, one has to cope with the problem of identifying and quantifying the external preferences attached to individuals. This is of course an open and difficult problem: a practical method would be to analyze historical positions of each individual on other topics (for example, in the context of misinformation [12], the attached bias may correspond to the frequency at which conspiracy theories has been preferred to mainstream news in the past, by the individual). Another approach would be to first identify communities in a social network of interactions and then infer the average preferences of the individuals of the communities by analyzing the opinion uploads during internal and external interactions (similarly to [48]).

Additionally, the bipopulated VMP presented in this work serves as a foundation for more sophisticated and realistic models. For example, preferences can be formulated to depend on the current opinions of the two groups. Moreover, the assumption of assigning the same preference to all individuals in a community can be relaxed to explore the impact of different distributions on the asymptotic state of the system. Furthermore, one can consider the presence of noise or other biases (e.g., the algorithmic one [15]) or consider comparable time-scales for personal preferences and opinion uploads.

Finally, the VMP can be compared to other models with similar settings (e.g., [33,49]), to study the roles of preferences and homophily in the opinion formation process through multiple perspectives and approaches, as encouraged by the authors of [50,51].

## Figures and Tables

**Table 1:** Binary-state models with homogeneous personal biases (preferences). Ising Glauber: Ising model with Glauber dynamics, VMMI: voter model with media interaction, PVM: partisanship voter model. The models are defined through the infection and recovery transition rates $F_{k,m}$ and $R_{k,m}$ for σ:−1→+1 and σ:+1→−1, respectively; the considered node has *k* neighbors, out of which *m* are in state +1. The strength of the bias is *h*, which corresponds to the external field in the Ising model, with temperature *T* and pairwise couplings *J*. For the voter models, h∈[0,1].

$F_{k,m}$	$R_{k,m}$	
Ising Glauber	$\frac{1}{1+e^{-\frac{2}{T}\left[h+J\left(2m-k\right)\right]}}$	$\frac{e^{-\frac{2}{T}\left[h+J\left(2m-k\right)\right]}}{1+e^{-\frac{2}{T}\left[h+J\left(2m-k\right)\right]}}$
VMMI	$\left(1-h\right)\frac{m}{k}+h$	$\left(1-h\right)\left(1-\frac{m}{k}\right)$
PVM	$\frac{m}{k}\left(\frac{1+h}{2}\right)$	$\left(1-\frac{m}{k}\right)\left(\frac{1-h}{2}\right)$

**Table 2:** Bipopulated binary-state models with personal biases (preferences). Columns two and three (four and five) are for nodes in population 1 (2). For the VMMI, $h_{1}\in \left[0,1\right]$ and $h_{2}\in \left[-1,0\right]$.

$F_{k,m}^{\left(1\right)}$	$R_{k,m}^{\left(1\right)}$	$F_{k,m}^{\left(2\right)}$	$R_{k,m}^{\left(2\right)}$	
Ising Glauber	$\frac{1}{1+e^{-\frac{2}{T}\left[h_{1}+J\left(2m-k\right)\right]}}$	$\frac{e^{-\frac{2}{T}\left[h_{1}+J\left(2m-k\right)\right]}}{1+e^{-\frac{2}{T}\left[h_{1}+J\left(2m-k\right)\right]}}$	$\frac{1}{1+e^{-\frac{2}{T}\left[h_{2}+J\left(2m-k\right)\right]}}$	$\frac{e^{-\frac{2}{T}\left[h_{2}+J\left(2m-k\right)\right]}}{1+e^{-\frac{2}{T}\left[h_{2}+J\left(2m-k\right)\right]}}$
VMMI	$\left(1-h_{1}\right)\frac{m}{k}+h_{1}$	$\left(1-h_{1}\right)\left(1-\frac{m}{k}\right)$	$\left(1-\right|h_{2}\left|\right)\frac{m}{k}$	$\left(1-\right|h_{2}\left|\right)\left(1-\frac{m}{k}\right)+\left|h_{2}\right|$
PVM	$\frac{m}{k}\left(\frac{1+h_{1}}{2}\right)$	$\left(1-\frac{m}{k}\right)\left(\frac{1-h_{1}}{2}\right)$	$\frac{m}{k}\left(\frac{1+h_{2}}{2}\right)$	$\left(1-\frac{m}{k}\right)\left(\frac{1-h_{2}}{2}\right)$

## Data Availability

Data on 2004 Political Blogosphere [42] can be found at www-personal.umich.edu/mejn/netdata/ (accessed on 5 April 2023).

## Abbreviations

The following abbreviations are used in this manuscript:

ODEs	Ordinary Differential Equations

## Appendix A. Linear Stability Analysis

### Appendix A.1. Fully Connected Network

Here, we perform the linear stability analysis of the dynamical system (4) derived for the VMP on the fully connected topology, giving a proof to the considerations in Section 3.1. As said in the main text, the consensus points (0,0) and (α,1−α) are fixed points of the system for whatever choice of the parameters $\alpha ,h_{1},h_{2}$, while it is easy to prove that only for $\alpha \in (\alpha_{c}^{-},\alpha_{c}^{+})$ the fixed point corresponding to the polarized states (5) and (6) is in the rectangle (0,α)×(0,1−α) and thus has a physical meaning. In the following, we prove that for conflicting preferences, i.e., $h_{1}\geq 0,h_{2}\leq 0$, for $\alpha <\alpha_{c}^{-}$ the negative consensus fixed point is the only stable fixed point of the system, for $\alpha \in (\alpha_{c}^{-},\alpha_{c}^{+})$ both the consensus points are unstable and the polarized fixed point is stable, while for $\alpha >\alpha_{c}^{-}$ the positive consensus is the only stable fixed point.

The Jacobian of the dynamical system reads

(A1) $$J\left(\rho_{1},\rho_{2};\alpha ,h_{1},h_{2}\right)=\frac{1}{2}\left(\begin{array}{cc} \alpha -1+h_{1}\left(1+\alpha -4\rho_{1}-2\rho_{2}\right) & \alpha +h_{1}\left(\alpha -2\rho_{1}\right)\\ 1-\alpha +h_{2}\left(1-\alpha -2\rho_{2}\right) & -\alpha +h_{2}\left(2-\alpha -4\rho_{2}-2\rho_{1}\right) \end{array}\right)$$

According to the linear stability theory, a fixed point is stable if both the eigenvalues of the corresponding Jacobian are negative, i.e., if the trace *T* is negative and the determinant *D* is positive.

For the negative consensus fixed point, we have that

(A2) $$\begin{array}{cc} \; & T=\frac{1}{2}\left[\alpha \left(h_{1}-h_{2}\right)+2h_{2}+h_{1}-1\right] \end{array}$$

(A3) $$\begin{array}{cc} \; & D=\frac{1}{2}\left[h_{1}h_{2}-h_{2}-\alpha \left(h_{1}-h_{2}\right)\right] \end{array}$$

so the determinant is positive for $\alpha <\alpha^{D}=\frac{-h_{2}\left(1-h_{1}\right)}{h_{1}-h_{2}}$, while the trace is negative for $\alpha <\alpha^{T}=\frac{1-2h_{2}-h_{1}}{h_{1}-h_{2}}$. Thus, the stability condition is satisfied for $\alpha <\min(\alpha^{T},\alpha^{D})$. It is easy to see that $\alpha^{D}\leq \alpha^{T}$, because for the assumptions on the sign of the preferences it holds that $h_{1}h_{2}+h_{1}+h_{2}\leq 1$, so the negative consensus point is a stable attractive fixed point if and only if $\alpha <\alpha^{D}=\alpha_{c}^{-}$.

Analogous considerations apply to the positive consensus fixed points, whose determinant and trace read

(A4) $$\begin{array}{cc} \; & T=\frac{1}{2}\left[-\alpha \left(h_{1}-h_{2}\right)-h_{1}-2h_{2}-1\right] \end{array}$$

(A5) $$\begin{array}{cc} \; & D=\frac{1}{2}\left[\alpha \left(h_{1}-h_{2}\right)+h_{1}h_{2}+h_{2}\right] \end{array}$$

and by applying the same arguments as before, we obtain that the stability condition is fulfilled when $\alpha >max\left(\alpha^{D},\alpha^{T}\right)=\alpha^{D}=\alpha_{c}^{+}$.

Last, the same considerations about the trace and determinant can be used to prove that in the range $\left[\alpha_{c}^{-},\alpha_{c}^{+}\right]$ the fixed point corresponding to the polarized state is stable.

### Appendix A.2. Modular Networks

We perform the linear stability analysis of the mean-field system (10) derived from the VMP on a modular network with two communities and open-mindedness parameters $\gamma_{1},\gamma_{2}$. The dynamical variables taken into consideration are now $\rho_{1}^{\prime },\rho_{2}^{\prime }$ as defined in Section 3.2, both in the range [0,1]. The analysis focuses on the stability of the consensus points, now (0,0) for the negative and (1,1) for the positive, which are fixed points of the system for whatever choice of the parameters.

The Jacobian matrix of the system (10), discarding the uninfluential factor, reads

(A6) $$J\left(\rho_{1}^{\prime },\rho_{2}^{\prime }\right)=\left(\begin{array}{cc} \gamma_{1}\left(4h_{1}\rho_{1}-2h_{1}\rho_{2}-h_{1}-1\right)-4h_{1}\rho_{1}+2h_{1} & \gamma_{1}\left[1-h_{1}\left(2\rho_{1}-1\right)\right]\\ \gamma_{2}\left[1-h_{2}\left(2\rho_{2}-1\right)\right] & \gamma_{2}\left(4h_{2}\rho_{2}-2h_{2}\rho_{1}-h_{2}-1\right)-4h_{2}\rho_{2}+2h_{2} \end{array}\right)$$

and as before, we compute the traces and the determinant of the Jacobian at the fixed point in order to obtain the stability conditions.

The traces and determinant for the positive consensus point read

(A7) $$\begin{array}{cc} \; & T=-\gamma_{1}\left(1-h_{1}\right)-\gamma_{2}\left(1-h_{2}\right)-2\left(h_{1}+h_{2}\right) \end{array}$$

(A8) $$\begin{array}{cc} \; & D=2\left[\gamma_{1}h_{2}\left(1-h_{1}\right)+\gamma_{2}h_{1}\left(1-h_{2}\right)+2h_{1}h_{2}\right] \end{array}$$

and we see that the trace is negative for all $h_{1}\geq -h_{2}$, while the determinant is positive for

(A9) $$\gamma_{1}h_{2}\left(1-h_{1}\right)+\gamma_{2}h_{1}\left(1-h_{2}\right)+2h_{1}h_{2}>0$$

However, it never happens that T>0 and D>0 at the same time, at least in the parameters’ space of interest $h_{1}\geq 0,h_{2}\leq 0,\gamma_{1/2}\geq 0$. To prove it, we try to solve the system

(A10) $$\left\{\begin{array}{c} T=-\gamma_{1}\left(1-h_{1}\right)-\gamma_{2}\left(1+\right|h_{2}|)-2(h_{1}-|h_{2}\left|\right)>0\\ D\propto -\gamma_{1}|h_{2}|\left(1-h_{1}\right)+\gamma_{2}h_{1}\left(1+\right|h_{2}\left|\right)-2h_{1}\left|h_{2}\right|>0 \end{array}\right.$$

Arranging the terms, we are left with the series of inequalities

(A11) $$-\gamma_{1}\left(1-h_{1}\right)-2(h_{1}-|h_{2}|)>\gamma_{2}\left(1+\right|h_{2}\left|\right)>\frac{1}{h_{1}}\left(\gamma_{1}|h_{2}|\left(1-h_{1}\right)+2h_{1}\left|h_{2}\right|\right)$$

that implies

(A12) $$-\gamma_{1}\left(1-h_{1}\right)-2(h_{1}-|h_{2}\left|\right)>\frac{1}{h_{1}}\left(\gamma_{1}|h_{2}|\left(1-h_{1}\right)+2h_{1}\left|h_{2}\right|\right)$$

and simplifies in

(A13) $$-\gamma_{1}h_{1}\left(1-h_{1}\right)-2h_{1}^{2}>\gamma_{1}\left|h_{2}\right|\left(1-h_{1}\right)$$

which is never true, because the terms on the l.h.s. are always negative and the term on the r.h.s. is positive. Thus, the whole stability region of the positive consensus fixed point is determined by the condition derived from the determinant (A9), and thus delimited by the critical curve

(A14) $$\gamma_{1}h_{2}\left(1-h_{1}\right)+\gamma_{2}h_{1}\left(1-h_{2}\right)+2h_{1}h_{2}=0$$

For the negative consensus, we have

(A15) $$\begin{array}{cc} \; & T=-\gamma_{1}\left(1+h_{1}\right)-\gamma_{2}\left(1+h_{2}\right)+2\left(h_{1}+h_{2}\right) \end{array}$$

(A16) $$\begin{array}{cc} \; & D=2\left[-\gamma_{1}h_{2}\left(1+h_{1}\right)-\gamma_{2}h_{1}\left(1+h_{2}\right)+2h_{1}h_{2}\right] \end{array}$$

and applying the same arguments of the positive consensus, we can claim that the stability condition is determined only by the condition on the determinant D>0; thus, the corresponding critical curve reads

(A17) $$-\gamma_{1}h_{2}\left(1+h_{1}\right)-\gamma_{2}h_{1}\left(1+h_{2}\right)+2h_{1}h_{2}=0$$

## Appendix B. Mean-Field Transition Rates for Modular Networks

As in the fully connected case, each of the global mean-field transition rates (7) for the modular network is the product of three factors: the probability to randomly select an agent of class *i* and current state σ, the probability of selecting one neighbor of such an agent’s type currently in the opposite state −σ and the probability of transition (imitation). With respect to the fully connected case, the first and the third factor are obviously unchanged and in the case of $R_{+1}$ correspond, respectively, to $\alpha -\rho_{1}$ and $\frac{1+h_{1}}{2}$. To determine the second factor, we have to take carefully into account the modular structure and distinguish the two classes. For $R_{+1}$, once a spin of the first class is selected, the probability of randomly selecting a neighboring agent of the first class is $\frac{\alpha p_{11}}{\alpha p_{11}+\left(1-\alpha \right)p_{12}}$, multiplied by the probability that such a neighbor is in the up state $\frac{\rho_{1}}{\alpha }$. Analogously, the probability of randomly selecting a neighbor of the second class is $\frac{\left(1-\alpha \right)p_{12}}{\alpha p_{11}+\left(1-\alpha \right)p_{12}}$, multiplied by the probability that such a neighbor is in the up state $\frac{\rho_{2}}{1-\alpha }$. The result is the factor $\frac{p_{11}\rho_{1}}{\alpha p_{11}+\left(1-\alpha \right)p_{12}}+\frac{p_{12}\rho_{2}}{\alpha p_{11}+\left(1-\alpha \right)p_{12}}$. Analogous considerations apply for the other transition rates $R_{1-},R_{2+}$ and $R_{2-}$.

## Appendix C. Derivation of the Pair Approximation System of ODEs

For the sake of simplicity, we apply the pair approximation on a z-regular undirected modular graph with two communities, but the treatment can be easily extended to a directed modular network with heterogeneous degrees.

Each node of class 1 has $z_{11}$ internal neighbors and $z_{12}$ external (of different class) ones. The same applies for class 2. Moreover, $N_{1}z_{12}=N_{2}z_{21}$. The following quantities are defined: the density of up spins in the first and second class are, respectively, $\rho_{1}^{\prime }$ and $\rho_{2}^{\prime },$ as in the paragraph before, while $b_{lm}^{\sigma_{i}\;\sigma_{j}}$ represents the fraction of active links connecting a spin in state $\sigma_{i}$ spin of class *l* with a spin in state $\sigma_{j}$ spin of class *m*, normalized by the total number of connections of the class *l* toward the class *m*, considering the adjacency matrix *A* associated to the network:

(A18) $$\begin{array}{cc} \; & \rho_{1}^{\prime }=\frac{1}{N_{1}}\sum_{i=1}^{N_{1}}\frac{1+\sigma_{i}}{2} \end{array}$$

(A19) $$\begin{array}{cc} \; & \rho_{2}^{\prime }=\frac{1}{N_{2}}\sum_{i=N_{1}+1}^{N}\frac{1+\sigma_{i}}{2} \end{array}$$

(A20) $$\begin{array}{cc} \; & b_{11}^{+-}=\frac{1}{N_{1}z_{11}}\sum_{i=1}^{N_{1}}\;\sum_{j=1}^{N_{1}}A_{ij}\frac{1-\sigma_{i}+\sigma_{j}-\sigma_{i}\sigma_{j}}{4} \end{array}$$

(A21) $$\begin{array}{cc} \; & b_{22}^{+-}=\frac{1}{N_{2}z_{22}}\sum_{i=N_{1}+1}^{N}\;\sum_{j=N_{1}+1}^{N}A_{ij}\frac{1-\sigma_{i}+\sigma_{j}-\sigma_{i}\sigma_{j}}{4} \end{array}$$

(A22) $$\begin{array}{cc} \; & b_{12}^{-+}=\frac{1}{N_{1}z_{12}}\sum_{i=1}^{N_{1}}\sum_{j=N_{1}+1}^{N}A_{ij}\frac{1-\sigma_{i}+\sigma_{j}-\sigma_{i}\sigma_{j}}{4} \end{array}$$

(A23) $$\begin{array}{cc} \; & b_{12}^{+-}=\frac{1}{N_{1}z_{12}}\sum_{i=1}^{N_{1}}\sum_{j=N_{1}+1}^{N}A_{ij}\frac{1+\sigma_{i}-\sigma_{j}-\sigma_{i}\sigma_{j}}{4} \end{array}$$

Due to the undirectedness of the network, the other quantities of interest can be expressed as functions of the ones defined above, specifically $b_{11}^{-+}=b_{11}^{+-}$, $b_{22}^{-+}=b_{22}^{+-}$, $b_{21}^{+-}=b_{12}^{-+}$, $b_{21}^{-+}=b_{12}^{+-}$.

The first of the global rates of the possible processes $W_{z_{11},m_{11},z_{12},m_{12}}^{1-\to +}$, i.e., the probability that in a unit time (recall $\delta t=\frac{1}{N}$) a spin of the first class in current state − and with $m_{11}$ out of $z_{11}$ internal neighbors and $m_{12}$ out of $z_{12}$ external ones currently in + state flips, reads

(A24) $$\begin{array}{cc} \; & W_{z_{11},m_{11},z_{12},m_{12}}^{1-\to +}=N_{1}\left(1-\rho_{1}^{\prime }\right)P_{z_{11},m_{11}}^{11-}P_{z_{12},m_{12}}^{12-}F_{z_{11}+z_{12},m_{11}+m_{12}}^{1} \end{array}$$

where $P_{z_{11},m_{11}}^{11-}$ is the probability that a node in the first population currently in state −1 has $z_{11}$ degree (trivial, explicitly stated only for the generalization) and $m_{11}$ neighbors of the first population currently in state +1. The other rates read similarly:

(A25) $$\begin{array}{cc} \; & W_{z_{22},m_{22},z_{21},m_{21}}^{2-\to +}=N_{2}\left(1-\rho_{2}^{\prime }\right)P_{z_{22},m_{22}}^{22-}P_{z_{21},m_{21}}^{21-}F_{z_{22}+z_{21},m_{22}+m_{21}}^{2} \end{array}$$

(A26) $$\begin{array}{cc} \; & W_{z_{11},m_{11},z_{12},m_{12}}^{1+\to -}=N_{1}\rho_{1}^{\prime }P_{z_{11},m_{11}}^{11+}P_{z_{12},m_{12}}^{12+}R_{z_{11}+z_{12},m_{11}+m_{12}}^{1} \end{array}$$

(A27) $$\begin{array}{cc} \; & W_{z_{22},m_{22},z_{21},m_{21}}^{2+\to -}=N_{2}\rho_{2}^{\prime }P_{z_{22},m_{22}}^{22+}P_{z_{21},m_{21}}^{21+}R_{z_{22}+z_{21},m_{22}+m_{21}}^{2} \end{array}$$

The transition rates (infection −1→+1 and recovery +1→−1) in the expressions of the global rates, for this specific multi-class binary-state stochastic process, are

(A28) $$\begin{array}{cc} \; & F_{z,m}^{1/2}=\frac{m}{z}\frac{1+h_{1/2}}{2} \end{array}$$

(A29) $$\begin{array}{cc} \; & R_{z,m}^{1/2}=\left(1-\frac{m}{z}\right)\frac{1-h_{1/2}}{2} \end{array}$$

The two global rates of the first class $W_{z_{11},m_{11},z_{12},m_{12}}^{1\mp \to \pm }$ change the variables, respectively,

(A30) $$\begin{array}{cc} \; & \rho_{1}^{\prime }\to \rho_{1}^{\prime }\pm \frac{1}{N_{1}} \end{array}$$

(A31) $$\begin{array}{cc} \; & b_{11}^{+-}\to b_{11}\pm \frac{\left(z_{11}-2m_{11}\right)}{N_{1}z_{11}} \end{array}$$

(A32) $$\begin{array}{cc} \; & b_{12}^{-+}\to b_{12}^{-+}\mp \frac{m_{12}}{N_{1}z_{12}} \end{array}$$

(A33) $$\begin{array}{cc} \; & b_{12}^{+-}\to b_{12}^{+-}\pm \frac{z_{12}-m_{12}}{N_{1}z_{12}} \end{array}$$

and correspondingly the ones in the second class rates $W_{z_{22},m_{22},z_{21},m_{21}}^{2\mp \to \pm }$ change the variables

(A34) $$\begin{array}{cc} \; & \rho_{2}^{\prime }\to \rho_{2}^{\prime }\pm \frac{1}{N_{2}} \end{array}$$

(A35) $$\begin{array}{cc} \; & b_{22}^{+-}\to b_{22}\pm \frac{\left(z_{22}-2m_{22}\right)}{N_{2}z_{22}} \end{array}$$

(A36) $$\begin{array}{cc} \; & b_{12}^{-+}\to b_{12}^{-+}\pm \frac{z_{21}-m_{21}}{N_{2}z_{21}} \end{array}$$

(A37) $$\begin{array}{cc} \; & b_{12}^{+-}\to b_{12}^{+-}\mp \frac{m_{21}}{N_{2}z_{21}} \end{array}$$

Thus, the dynamical system consists of six coupled evolution equations:

(A38) $$\left\{\begin{array}{c} \dot{\rho_{1}^{\prime }}=\frac{1}{N_{1}}\sum_{m_{11}=0}^{z_{11}}\sum_{m_{12}=0}^{z_{12}}\left[W_{z_{11},m_{11},z_{12},m_{12}}^{1+}-W_{z_{11},m_{11},z_{12},m_{12}}^{1-}\right]\\ \dot{\rho_{2}^{\prime }}=\frac{1}{N_{2}}\sum_{m_{22}=0}^{z_{22}}\sum_{m_{21}=0}^{z_{21}}\left[W_{z_{22},m_{22},z_{21},m_{21}}^{2+}-W_{z_{22},m_{22},z_{21},m_{21}}^{2-}\right]\\ \dot{b_{11}^{+-}}=\frac{1}{N_{1}z_{11}}\sum_{m_{11}=0}^{z_{11}}\sum_{m_{12}=0}^{z_{12}}\left(z_{11}-2m_{11}\right)\left[W_{z_{11},m_{11},z_{12},m_{12}}^{1+}-W_{z_{11},m_{11},z_{12},m_{12}}^{1-}\right]\\ \dot{b_{22}^{+-}}=\frac{1}{N_{2}z_{22}}\sum_{m_{22}=0}^{z_{22}}\sum_{m_{21}=0}^{z_{21}}\left(z_{22}-2m_{22}\right)\left[W_{z_{22},m_{22},z_{21},m_{21}}^{2+}-W_{z_{22},m_{22},z_{21},m_{21}}^{2-}\right]\\ \dot{b_{12}^{-+}}=\frac{1}{N_{1}z_{12}}\sum_{m_{11}=0}^{z_{11}}\sum_{m_{12}=0}^{z_{12}}\left(-m_{12}\right)\left[W_{z_{11},m_{11},z_{12},m_{12}}^{1+}-W_{z_{11},m_{11},z_{12},m_{12}}^{1-}\right]\;+\\ \;\;\;\;\;\;\;\;\;\;\;\;\;+\frac{1}{N_{2}z_{21}}\sum_{m_{22}=0}^{z_{22}}\sum_{m_{21}=0}^{z_{21}}\left(z_{21}-m_{21}\right)\left[W_{z_{22},m_{22},z_{21},m_{21}}^{2+}-W_{z_{22},m_{22},z_{21},m_{21}}^{2-}\right]\\ \dot{b_{12}^{+-}}=\frac{1}{N_{1}z_{12}}\sum_{m_{11}=0}^{z_{11}}\sum_{m_{12}=0}^{z_{12}}\left(z_{12}-m_{12}\right)\left[W_{z_{11},m_{11},z_{12},m_{12}}^{1+}-W_{z_{11},m_{11},z_{12},m_{12}}^{1-}\right]\;+\\ \;\;\;\;\;\;\;\;\;\;\;\;\;+\frac{1}{N_{2}z_{21}}\sum_{m_{22}=0}^{z_{22}}\sum_{m_{21}=0}^{z_{21}}\left(-m_{21}\right)\left[W_{z_{22},m_{22},z_{21},m_{21}}^{2+}-W_{z_{22},m_{22},z_{21},m_{21}}^{2-}\right] \end{array}\right.$$

We still have to define the probabilities, e.g., $P_{z_{11},m_{11}}^{11-}$, whose definition would close the system of ordinary differential equations above.

The mean-field approximation corresponds to taking the probabilities

(A39) $$\begin{array}{cc} \; & P_{z_{11},m_{11}}^{11+}=P_{z_{11},m_{11}}^{11-}=\delta \left(\frac{m_{11}}{z_{11}}-\rho_{1}^{\prime }\right) \end{array}$$

(A40) $$\begin{array}{cc} \; & P_{z_{22},m_{22}}^{22+}=P_{z_{22},m_{22}}^{22-}=\delta \left(\frac{m_{22}}{z_{22}}-\rho_{2}^{\prime }\right) \end{array}$$

(A41) $$\begin{array}{cc} \; & P_{z_{12},m_{12}}^{12+}=P_{z_{12},m_{12}}^{12-}=\delta \left(\frac{m_{12}}{z_{12}}-\rho_{2}^{\prime }\right) \end{array}$$

(A42) $$\begin{array}{cc} \; & P_{z_{21},m_{21}}^{21+}=P_{z_{21},m_{21}}^{21-}=\delta \left(\frac{m_{21}}{z_{21}}-\rho_{1}^{\prime }\right) \end{array}$$

and thus decoupling the evolution of the densities of the up spin from the rest of the system; indeed, by inserting those probabilities we would recover the mean-field evolution Equation (10) for $\rho_{1}^{\prime },\rho_{2}^{\prime }$.

The pair approximation, instead, consists of assuming that those probabilities are binomial distributions Bz,m(x)=zmxm(1−x)z−m

(A43) $$\begin{array}{cc} \; & P_{z_{11},m_{11}}^{11-}=B_{z_{11},m_{11}}\left(p_{11-}\right) \end{array}$$

(A44) $$\begin{array}{cc} \; & P_{z_{11},m_{11}}^{11+}=B_{z_{11},z_{11}-m_{11}}\left(p_{11+}\right) \end{array}$$

(A45) $$\begin{array}{cc} \; & P_{z_{22},m_{22}}^{22-}=B_{z_{22},m_{22}}\left(p_{22-}\right) \end{array}$$

(A46) $$\begin{array}{cc} \; & P_{z_{22},m_{22}}^{22+}=B_{z_{22},z_{22}-m_{22}}\left(p_{22+}\right) \end{array}$$

(A47) $$\begin{array}{cc} \; & P_{z_{12},m_{12}}^{12-}=B_{z_{12},m_{12}}\left(p_{12-}\right) \end{array}$$

(A48) $$\begin{array}{cc} \; & P_{z_{12},m_{12}}^{12+}=B_{z_{12},z_{12}-m_{12}}\left(p_{12+}\right) \end{array}$$

(A49) $$\begin{array}{cc} \; & P_{z_{21},m_{21}}^{21-}=B_{z_{21},m_{21}}\left(p_{21-}\right) \end{array}$$

(A50) $$\begin{array}{cc} \; & P_{z_{21},m_{21}}^{21+}=B_{z_{21},z_{21}-m_{21}}\left(p_{21+}\right) \end{array}$$

with single event probabilities

(A51) $$\begin{array}{cc} \; & p_{11-}=\frac{b_{11}^{-+}}{1-\rho_{1}^{\prime }}=\frac{b_{11}^{+-}}{1-\rho_{1}^{\prime }} \end{array}$$

(A52) $$\begin{array}{cc} \; & p_{11+}=\frac{b_{11}^{+-}}{\rho_{1}^{\prime }} \end{array}$$

(A53) $$\begin{array}{cc} \; & p_{22-}=\frac{b_{22}^{-+}}{1-\rho_{2}^{\prime }}=\frac{b_{22}^{+-}}{1-\rho_{2}^{\prime }} \end{array}$$

(A54) $$\begin{array}{cc} \; & p_{22+}=\frac{b_{22}^{+-}}{\rho_{2}^{\prime }} \end{array}$$

(A55) $$\begin{array}{cc} \; & p_{12-}=\frac{b_{12}^{-+}}{1-\rho_{1}^{\prime }} \end{array}$$

(A56) $$\begin{array}{cc} \; & p_{12+}=\frac{b_{12}^{+-}}{\rho_{1}^{\prime }} \end{array}$$

(A57) $$\begin{array}{cc} \; & p_{21-}=\frac{b_{21}^{+-}}{1-\rho_{2}^{\prime }}=\frac{b_{12}^{-+}}{1-\rho_{2}^{\prime }} \end{array}$$

(A58) $$\begin{array}{cc} \; & p_{21+}=\frac{b_{21}^{+-}}{\rho_{2}^{\prime }}=\frac{b_{12}^{-+}}{\rho_{2}^{\prime }} \end{array}$$

The criterion is to consider as a single probability, e.g., to express $p_{11-}$, the fraction of −+ links of the first communities (in number $b_{11}^{-+}N_{1}z_{11}$) over the total of the edges starting from − within the first community (in number $\left(1-\rho_{1}^{\prime }\right)N_{1}z_{11}$).

Eventuallym we can write the final system of the ODE within the pair approximation

(A59) $$\left\{\begin{array}{c} \dot{\rho_{1}^{\prime }}=\left(1-\rho_{1}^{\prime }\right)\sum_{m_{11}=0}^{z_{11}}B_{z_{11},m_{11}}\left(p_{11-}\right)\sum_{m_{12}=0}^{z_{12}}B_{z_{12},m_{12}}\left(p_{12-}\right)F_{z_{11}+z_{12},m_{11}+m_{12}}^{1}+\\ \;\;\;\;\;\;\;\;\;-\rho_{1}^{\prime }\sum_{m_{11}=0}^{z_{11}}B_{z_{11},z_{11}-m_{11}}\left(p_{11+}\right)\sum_{m_{12}=0}^{z_{12}}B_{z_{12},z_{12}-m_{12}}\left(p_{12+}\right)R_{z_{11}+z_{12},m_{11}+m_{12}}^{1}\\ \dot{\rho_{2}^{\prime }}=\left(1-\rho_{2}^{\prime }\right)\sum_{m_{22}=0}^{z_{22}}B_{z_{22},m_{22}}\left(p_{22-}\right)\sum_{m_{21}=0}^{z_{21}}B_{z_{21},m_{21}}\left(p_{21-}\right)F_{z_{22}+z_{21},m_{22}+m_{21}}^{2}+\\ \;\;\;\;\;\;\;\;\;-\rho_{2}^{\prime }\sum_{m_{22}=0}^{z_{22}}B_{z_{22},z_{22}-m_{22}}\left(p_{22+}\right)\sum_{m_{21}=0}^{z_{21}}B_{z_{21},z_{21}-m_{21}}\left(p_{21+}\right)R_{z_{22}+z_{21},m_{22}+m_{21}}^{2}\\ \dot{b_{11}^{+-}}=\frac{1-\rho_{1}^{\prime }}{z_{11}}\sum_{m_{11}=0}^{z_{11}}\sum_{m_{12}=0}^{z_{12}}\left(z_{11}-2m_{11}\right)B_{z_{11},m_{11}}\left(p_{11-}\right)B_{z_{12},m_{12}}\left(p_{12-}\right)F_{z_{11}+z_{12},m_{11}+m_{12}}^{1}+\\ \;\;\;\;\;\;\;\;\;\;-\frac{\rho_{1}^{\prime }}{z_{11}}\sum_{m_{11}=0}^{z_{11}}\sum_{m_{12}=0}^{z_{12}}\left(z_{11}-2m_{11}\right)B_{z_{11},z_{11}-m_{11}}\left(p_{11+}\right)B_{z_{12},z_{12}-m_{12}}\left(p_{12+}\right)R_{z_{11}+z_{12},m_{11}+m_{12}}^{1}\\ \dot{b_{22}^{+-}}=\frac{1-\rho_{2}^{\prime }}{z_{22}}\sum_{m_{22}=0}^{z_{22}}\sum_{m_{21}=0}^{z_{21}}\left(z_{22}-2m_{22}\right)B_{z_{22},m_{22}}\left(p_{22-}\right)B_{z_{21},m_{21}}\left(p_{21-}\right)F_{z_{22}+z_{21},m_{22}+m_{21}}^{2}+\\ \;\;\;\;\;\;\;\;\;-\frac{\rho_{2}^{\prime }}{z_{22}}\sum_{m_{22}=0}^{z_{22}}\sum_{m_{21}=0}^{z_{21}}\left(z_{22}-2m_{22}\right)B_{z_{22},z_{22}-m_{22}}\left(p_{22+}\right)B_{z_{21},z_{21}-m_{21}}\left(p_{21+}\right)R_{z_{22}+z_{21},m_{22}+m_{21}}^{2}\\ \dot{b_{12}^{-+}}=\frac{1-\rho_{1}^{\prime }}{z_{12}}\sum_{m_{11}=0}^{z_{11}}\sum_{m_{12}=0}^{z_{12}}\left(-m_{12}\right)B_{z_{11},m_{11}}\left(p_{11-}\right)B_{z_{12},m_{12}}\left(p_{12-}\right)F_{z_{11}+z_{12},m_{11}+m_{12}}^{1}+\\ \;\;\;\;\;\;\;\;\;\;-\frac{\rho_{1}^{\prime }}{z_{12}}\sum_{m_{11}=0}^{z_{11}}\sum_{m_{12}=0}^{z_{12}}\left(-m_{12}\right)B_{z_{11},z_{11}-m_{11}}\left(p_{11+}\right)B_{z_{12},z_{12}-m_{12}}\left(p_{12+}\right)R_{z_{11}+z_{12},m_{11}+m_{12}}^{1}+\\ \;\;\;\;\;\;\;\;\;\;+\frac{1-\rho_{2}^{\prime }}{z_{21}}\sum_{m_{22}=0}^{z_{22}}\sum_{m_{21}=0}^{z_{21}}\left(z_{21}-m_{21}\right)B_{z_{22},m_{22}}\left(p_{22-}\right)B_{z_{21},m_{21}}\left(p_{21-}\right)F_{z_{22}+z_{21},m_{22}+m_{21}}^{2}+\\ \;\;\;\;\;\;\;\;\;\;-\frac{\rho_{2}^{\prime }}{z_{21}}\sum_{m_{22}=0}^{z_{22}}\sum_{m_{21}=0}^{z_{21}}\left(z_{21}-m_{21}\right)B_{z_{22},z_{22}-m_{22}}\left(p_{22+}\right)B_{z_{21},z_{21}-m_{21}}\left(p_{21+}\right)R_{z_{22}+z_{21},m_{22}+m_{21}}^{2}\\ \dot{b_{12}^{+-}}=\frac{1-\rho_{1}^{\prime }}{z_{12}}\sum_{m_{11}=0}^{z_{11}}\sum_{m_{12}=0}^{z_{12}}\left(z_{12}-m_{12}\right)B_{z_{11},m_{11}}\left(p_{11-}\right)B_{z_{12},m_{12}}\left(p_{12-}\right)F_{z_{11}+z_{12},m_{11}+m_{12}}^{1}+\\ \;\;\;\;\;\;\;\;\;\;-\frac{\rho_{1}^{\prime }}{z_{12}}\sum_{m_{11}=0}^{z_{11}}\sum_{m_{12}=0}^{z_{12}}\left(z_{12}-m_{12}\right)B_{z_{11},z_{11}-m_{11}}\left(p_{11+}\right)B_{z_{12},z_{12}-m_{12}}\left(p_{12+}\right)R_{z_{11}+z_{12},m_{11}+m_{12}}^{1}+\\ \;\;\;\;\;\;\;\;\;\;+\frac{1-\rho_{2}^{\prime }}{z_{21}}\sum_{m_{22}=0}^{z_{22}}\sum_{m_{21}=0}^{z_{21}}\left(-m_{21}\right)B_{z_{22},m_{22}}\left(p_{22-}\right)B_{z_{21},m_{21}}\left(p_{21-}\right)F_{z_{22}+z_{21},m_{22}+m_{21}}^{2}+\\ \;\;\;\;\;\;\;\;\;\;-\frac{\rho_{2}^{\prime }}{z_{21}}\sum_{m_{22}=0}^{z_{22}}\sum_{m_{21}=0}^{z_{21}}\left(-m_{21}\right)B_{z_{22},z_{22}-m_{22}}\left(p_{22+}\right)B_{z_{21},z_{21}-m_{21}}\left(p_{21+}\right)R_{z_{22}+z_{21},m_{22}+m_{21}}^{2} \end{array}\right.$$

to be solved numerically with standard methods.

From the initial conditions $\rho_{1}^{\prime }\left(0\right),\rho_{2}^{\prime }\left(0\right)$, the other initial conditions are determined as follows:

(A60) $$\begin{array}{cc} \; & b_{11}^{+-}\left(0\right)=\rho_{1}^{\prime }\left(0\right)\left(1-\rho_{1}^{\prime }\left(0\right)\right) \end{array}$$

(A61) $$\begin{array}{cc} \; & b_{22}^{+-}\left(0\right)=\rho_{2}^{\prime }\left(0\right)\left(1-\rho_{2}^{\prime }\left(0\right)\right) \end{array}$$

(A62) $$\begin{array}{cc} \; & b_{12}^{-+}\left(0\right)=\rho_{1}^{\prime }\left(0\right)\left(1-\rho_{2}^{\prime }\left(0\right)\right) \end{array}$$

(A63) $$\begin{array}{cc} \; & b_{12}^{+-}\left(0\right)=\rho_{2}^{\prime }\left(0\right)\left(1-\rho_{1}^{\prime }\left(0\right)\right) \end{array}$$
